# Dangers from Protracted Inhalation of Ether

**Published:** 1888-05

**Authors:** 


### DANGERS FROM PROTRACTED INHALATION OF ETHER.

W. Gill Wylie, M. D., in the *Medical Record of March* 31, 1888, presented to the profession a summary of a year's work in abdominal surgery. The record is brilliant; out of forty-one operations for the removal of the uterine appendages but one death occurred, and that was from acute interstitial nephritis. Of seventeen ovariotomies, one died from suppression of urine, on the fourth day. The autopsy revealed chronic and acute diffused nephritis, with cirrhosis of the liver. One case of supra-pubic hysterectomy died of exhaustion, while two laparotomies, one for extra-uterine pregnancy, and the other for septic pelvic abscess, resulted fatally.

From the foregoing it appears that in two of the cases death was caused by inflammation of the kidneys. Dr. Wylie attributes the death of one of the patients to prolonged use of ether in the operation. This emphasizes the known dangers of ether inhalation, particularly in prolonged operations, in persons suffering from chronic interstitial nephritis. For years ether has been considered in the United States the anæsthetic *par excellence* so far as safety is concerned. Those appaling accidents in which a patient dies on the operating table were comparatively infrequent, and the *mortality* of ether as compared with chloroform was roughly estimated at from one to fifteen. The *nexus* between kidney complications and the inhalation of ether has been too often overlooked, and yet the records show many fatalities, in which the findings at the post-mortem table are not different from those in Dr. Wylie's cases. It is possible that, could the records be made up completely, the mortality from ether and chloroform in prolonged operations would vary less.

The practical lesson is that we should *never*, in such cases, administer an anæsthetic without first carefully examining the heart and urine. The latter should be especially examined for traces of albumen; but of more importance than that is the estimation of the total solids excreted by the urine. Of course tube-casts, or other evidence of gross kidney lesion, should at once contra-indicate the use of ether. The estimation of the quantity and solids of the urine furnishes the true index of the integrity of the renal structures and of their capacity to bear any added strain.

It may be argued that this is a troublesome and complicated procedure. But in these days when the mortality from surgical operations has reached such a diminishing point, the proverbial "one chance in a hundred" cannot afford to be missed by the enterprising surgeon.

Pneumonia is a sequela of ether-inhalation by no means infrequent, and one of those which readily escape estimation in tables illustrating the comparative safety of different anæsthetics.

